# Prolactin inhibitor changes testosterone production, testicular morphology, and related genes expression in cashmere goats

**DOI:** 10.3389/fvets.2023.1249189

**Published:** 2023-10-26

**Authors:** Meijing Chen, Chunhui Duan, Xuejiao Yin, Xianglong Li, Xiaona Liu, Lechao Zhang, Sicong Yue, Yingjie Zhang, Yueqin Liu

**Affiliations:** ^1^College of Animal Science and Technology, Hebei Agricultural University, Baoding, China; ^2^College of Animal Science and Technology, Hebei Normal University of Science and Technology, Qinhuangdao, China

**Keywords:** prolactin, testis, RNA-Seq, mRNA, steroid hormone biosynthesis

## Abstract

Prolactin has multifaceted roles in lactation, growth, metabolism, osmoregulation, behavior, and the reproduction of animals. This study aimed to investigate the involvement of prolactin in testicular function in cashmere goats. Twenty cashmere goats were randomly assigned to either the control group (CON) or the bromocriptine treatment group (BCR, bromocriptine, prolactin inhibitor). Blood and testis samples collected for analysis after 30 days of treatment. The results indicated that, compared with the CON group, BCR significantly decreased (*p* < 0.05) the serum concentrations of prolactin, and significantly increased (*p* < 0.05) the levels of testosterone and luteinizing hormone (LH) on day 30. The serum level of the follicle-stimulating hormone (FSH) was not affected (*p* > 0.05) by the treatment. The mean seminiferous tubule diameter and spermatogenic epithelium thickness were increased (*p* < 0.05) in the BCR group. Subsequently, we performed RNA sequencing and bioinformatics analysis to identify the key genes and pathways associated with the regulation of spermatogenesis or testosterone secretion function. A total of 142 differentially expressed genes (DEGs) were identified (91 were upregulated, 51 were downregulated). Gene ontology (GO) and Kyoto Encyclopedia of Genes and Genomes (KEGG) revealed that the DEGs were mainly involved in the extracellular matrix (ECM), hippo, and steroid hormone biosynthesis, which are related to testicular function. The expression of the genes *SULT2B1*, *CYP3A24*, and *CYP3A74* in the steroid hormone biosynthesis pathway significantly increased (*p* < 0.05) in the BCR group, which was validated by qRT-PCR. These results provide a basis for understanding the mechanisms underlying the regulation of testicular function by prolactin in cashmere goats.

## 1. Introduction

Spermatogenesis and testosterone production are major functions of the testes (1). Normal testicular function relies on endocrine and paracrine hormonal pathways (2), including follicle-stimulating hormone (FSH), luteinising hormone (LH), and testosterone (3). Prolactin (PRL) is a polypeptide hormone involved in various biological functions, such as lactation, growth performance, animal behavior, metabolism, and reproduction—and acts in conjunction with FSH and LH to regulate testicular function in adult male rats, hamsters, and mice (4, 5). PRL is mainly synthesized and secreted by the lactotroph cells of the anterior pituitary gland and acts *via* its membrane PRL receptor (PRLR) (6). PRLR has been identified in the testes of various species, including rams (7), rats (8), yaks (9), and humans (4). Studies have revealed that the PRLR in ram testes is expressed by Leydig cells and germ cells in seminiferous tubules, and the expression site and pattern of the receptor gene indicates a crucial role for PRL in the regulation of steroidogenesis and spermatogenesis (7).

Several studies have shown that the abnormal level of PRL affects the reproductive function. Hyperprolactinemia, in which circulating PRL levels are higher than those in the reference population, may result in decreased sperm production, and infertility in men (10); and lead to decreased LH and FSH levels in rats (11). Besides, clinical observations in hypoprolactinemic infertile men have shown that the restoration of normal PRL levels leads to an increase in sperm density and quality and restores fertility, suggesting a role for PRL in regulating the testis and accessory glands (12). Bromocriptine (BCR), a dopamine receptor agonist widely used to study the function of PRL, can reduce the concentration of PRL in mammals blood (13). A study on rams (*Ovis aries*) suggested that testicular function is influenced by BCR-induced hypoprolactinaemia (14). However, another study showed that testicular function and fertility were not affected in PRLR-knockout mice (15). Suppressing of the concentration of PRL using BCR decreases testicular volume, sperm production, and testosterone secretion in ram (16). A study on adult male rats illustrated that serum PRL level suppressed by 2-bromo-α-ergocriptine reduces the weight of the reproductive organ, but increases the serum level of LH, while FSH remains unaffected (17). Despite this, the pathways through which PRL regulates the function of the testes remain unclear. It is also unclear if PRL levels affect testosterone secretion and fertility in male goats.

In the present study, we hypothesized that PRL can change the serum hormone levels and the expression of related genes to influence the reproductive function. To test this hypothesis, we investigated the effects of PRL inhibition on serum biochemical indicators, testicular morphological, and differentially expressed mRNAs using transcriptome sequencing, and we try to elucidate the molecular mechanisms underlying PRL regulation of the testes in male cashmere goats.

## 2. Materials and methods

### 2.1. Ethics statement

This study was conducted under the guidance of the Animal Care and Use Committee of the Hebei Agricultural University (approval number: 2023133).

### 2.2. Animals and experiments

The study was conducted at the Qinglong Lihong Cashmere Goat Farm (Qinhuangdao, China) from August 17, 2020 to October 1, 2020. All goats had free access to fresh water and were fed twice daily (07,00 and 15,00 h) throughout the experiment, consistent with the requirements for raising and managing farms goats. All goats were housed in individual pens. Twenty healthy male goats (*Capra hircus*, Yanshan Cashmere goat breed, 10 months old, body weight = 22.98 ± 1.95 kg) were selected, and randomly assigned to two groups: 1) BCR treatment (*n* = 10, 0.06 mg/kg BW) and 2) the control group (CON, *n* = 10; equal volume of water). BCR was administered in the form of tablets dissolved in water that was sprayed onto the concentrate feed in the morning feed. The dose was based on the dosing instructions given in Zhang et al. (13) and Dicks et al. (18), which were approximately 0.05 mg/kg BW–0.07 mg/kg BW). The experiment was conducted over 45 days, with a 15-day adaptation period.

### 2.3. Sample collection

The body weights of the goats were recorded on days 0 and 30 before the morning feed, and the average daily gain (ADG) was calculated.

Before the morning feed was given, blood samples were obtained by jugular venipuncture from each goat into 5 mL coagulation-promoting tubes on days 0, 15, and 30. The samples were immediately centrifuged at 3000 × g for 15 min to harvest serum and stored at −20°C until analysis.

On day 30 of treatment, all goats were slaughtered at a local slaughterhouse before morning feeding to collect the testes. The left testes were immersed in 10% formaldehyde for histological analysis. A portion of the testicular samples from the right testis was immediately frozen in liquid nitrogen and then stored in a refrigerator at −80°C for subsequent RNA and protein extraction.

### 2.4. Hormone analysis

Serum concentrations of PRL, testosterone, FSH, and LH were determined using commercial goat enzyme-linked immunosorbent assay (ELISA) kits (Nanjing jiancheng Bio, Nanjing, China), in accordance with the manufacturer’s instructions. The absorbance (OD) of each well was measured at 450 nm and a standard curve was generated. Based on the standard curve, the concentrations of each sample were calculated as ng/mL, ng/L, mIU/mL, and mIU/mL, respectively.

### 2.5. Testicular morphological evaluation

The excised testes were fixed in 10% formaldehyde and kept at room-temperature overnight. The fixed tissues were dehydrated by automatic dewatering machine in ascending concentrations of graded ethanol baths (75, 85, 95 and 100%). Then the tissues were infiltrated with paraffin. The testicular tissues were sectioned into 5-μm-thick sections. After wax removal, the slides were stained with hematoxylin and eosin (H&E) and sealed with neutral gum. We randomly selected eight samples from each group and each slide was analyzed in at least five different fields. The diameters of the seminiferous tubules and the thickness of the spermatogenic epithelium were captured and measured using a Panoramic 250 digital microscope (3DHISTECH, Budapest, Hungary) at × 400 magnifications.

### 2.6. RNA isolation and evaluation of RNA integrity

The total RNA from each testis sample was extracted using TRIzol reagent (Invitrogen, Carlsbad, CA, United States) according to the manufacturer’s instructions. The RNA sample was qualified using 1% agarose gel electrophoresis for possible contamination and degradation. Thereafter, RNA purity, concentration, integrity, and quantity were examined and measured using the NanoPhotometer® spectrophotometer and RNA Nano 6,000 Assay Kit of the Bioanalyzer 2,100 system, respectively.

### 2.7. Library preparation, RNA sequencing, and data analysis

To create the library, 3 mg of high-quality RNA from each sample and the NEBNext Ultra Directional RNA Library Prep Kit for Illumina (NEB E7420) were used. Ribosomal RNA (rRNA) depletion and stranded method were used for the RNA library (19). After library construction, the concentration of the library was measured by the Qubit®fluorometer and adjusted to 1 ng/μL. An Agilent 2,100 Bioanalyzer was used to examine the library insert size. The qualified libraries were pooled and sequenced on Illumina platforms using the PE150 (paired-end 150 nt) strategy at Novogene Bioinformatics Technology Co., Ltd. (Beijing, China). Raw data (raw reads) in the FASTQ format were first processed using in-house Perl scripts. Further, clean data were screened from the raw reads by trimming and filtering reads containing adaptor, more than 10% unknown nucleotides (N), and more than 50% nucleotides with Qphred ≤20. Simultaneously, the Q20, Q30, and GC contents of the clean data was calculated. All downstream analyzes were performed based on high-quality clean data and were mapped to the *Capra hircus* reference genome using HISAT2 (20).

### 2.8. Differential expression and functional enrichment analysis of mRNAs

Gene quantification was performed using StringTie software and fragments per kilobase of transcript sequence per millions mapped reads (FPKM) were obtained. Differential expression was analyzed using DESeq2 (21), and the threshold of adjusted *p*-value <0.05 and |log2(fold change)| ≥ 1 were considered differentially expressed genes (DEGs) in CON vs. BCR. Furthermore, we performed gene ontogeny (GO) annotation of the DEGs using the GOseq R package and Kyoto Encyclopedia of Genes and Genomes (KEGG) enrichment analysis using the KEGG orthology-based annotation system (KOBAS).

### 2.9. Quantitative real-time PCR (qRT-PCR)

To verify the accuracy of the RNA-Seq, 14 differential expression (DE) mRNAs were randomly selected for qRT-PCR. Total RNA was extracted from tissues using TRIzol reagent (Invitrogen, Carlsbad, CA, United States) according to the manufacturer’s instructions, and reverse transcribed to cDNAs using an Evo M-MLV RT Kit with gDNA Eraser for qRT-PCR (Accurate, Hunan, China). qRT-PCR was performed using the SYBR Green Master Mix (Vazyme, Nanjing, China) and conducted on an ABI QuantStudio 7 Flex System. The qRT-PCR was performed in accordance with the following procedures: 95°C for 3 min, followed by 40 two-step amplification cycles of 95°C for 10 s and 60°C for 30 s. The primer sequences are listed in Table 1. The relative expression levels were computed by the 2^−ΔΔCt^ approach, with β-actin as an endogenous reference gene.

**Table 1:** List of the primers used in qRT-PCR.

Primer name	Sequence (5′-3′)	Amplicon size (bp)
TBXAS1-F	ACTTAGCGTTTTTCCGCCAG	206
TBXAS1-R	ACTGTCAGCCACTGGTTTGG	
CYP3A74-F	ACATTGCTGTCTCCAACCTTCACC	113
CYP3A74-R	GTGCCTTTCTCTGCTTCCTTCCTC	
COL11A2-F	ACTACATTCCGCCCTGGACT	158
COL11A2-R	TGGCCTGTACCTTAGGATGC	
SERPINA12-F	ATGGACGAGAAGGGCACAGAGG	95
SERPINA12-R	AGGAAGCGGCGGTTTATCTTGAC	
SULT2B1-F	CTCCAAGATCGCCAGGCAGTTG	122
SULT2B1-R	TCATCCGAATCCAGCCCTTAATGTG	
WEE2-F	AGAGGTCAGGATTCAGAGGCGAAG	85
WEE2-R	CTTTCCCGAAGTGTGCTGAGGTC	
CYP3A24-F	GCTGTGACGGTGCCAATCTCTG	147
CYP3A24-R	ATTTCGGGGTCCAGTTCCAAAAGG	
PKP2-F	CACACAGCGAGCACCAGTACAG	115
PKP2-R	ACAATTTCTGAGCGGGCGTAGC	
BANF2-F	GAGGGAAGCCGAGTTTCAGAAGTG	109
BANF2-R	CTACAGGAAGCAGGAGCACCATTC	
ALDH1A3-F	AGCAGCAATTTCTTCTCACCCTCAG	81
ALDH1A3-R	GCCTCCTTAACCAGCTTCCCAAC	
SV2C-F	TCTCTTGCCTCCTCTTGACTCTCG	92
SV2C-R	AGCCAGCACAGCATCACATTCC	
RPSO2-F	TAGAGGCCGTTGCTTTGAGG	184
RPSO2-R	GCCAACCTCACATCCCTTCCA	
PDCD1-F	ATGCCACCATTGTCTTCCCA	196
PDCD1-R	CCTTCTCCTCTCCACCACAC	
MVP-F	GGTCGGGCCAAAGACTTACA	111
MVP-R	GATCTCTAGGTCCGCATGGC	
β-actin-F	CCCTGGAGAAGAGCTACGAG	98
β-actin-R	CAGGAAGGAAGGCTGGAAGA	

### 2.10. Statistical analysis

All experiments were performed in triplicate, and the statistical significance between the two groups was evaluated by Student’s t-test using SPSS (version 21.0; SPSS, Chicago, United States). Data are presented as mean ± standard error of the mean. *p* < 0.05 was regarded as statistically significant.

## 3. Results

### 3.1. Body weight of cashmere goat

The body weights of the cashmere goats in the two groups are presented in Table 2. BCR treatment did not significantly affect (*p* > 0.05) the body weight or ADG of goats.

**Table 2:** Effects of bromocriptine on the growth performance of male cashmere goat.

Item^a^	CON	BCR	SEM	*p*-value
Initial weight, kg	22.39	23.57	1.952	0.563
Final weight, kg	23.20	24.25	1.885	0.593
Average daily gain, g	35.30	29.74	17.437	0.758

a CON, the control group; BCR, the bromocriptine treatment group.

### 3.2. Serum concentrations of PRL, testosterone, FSH, and LH level

**Figure 1:**
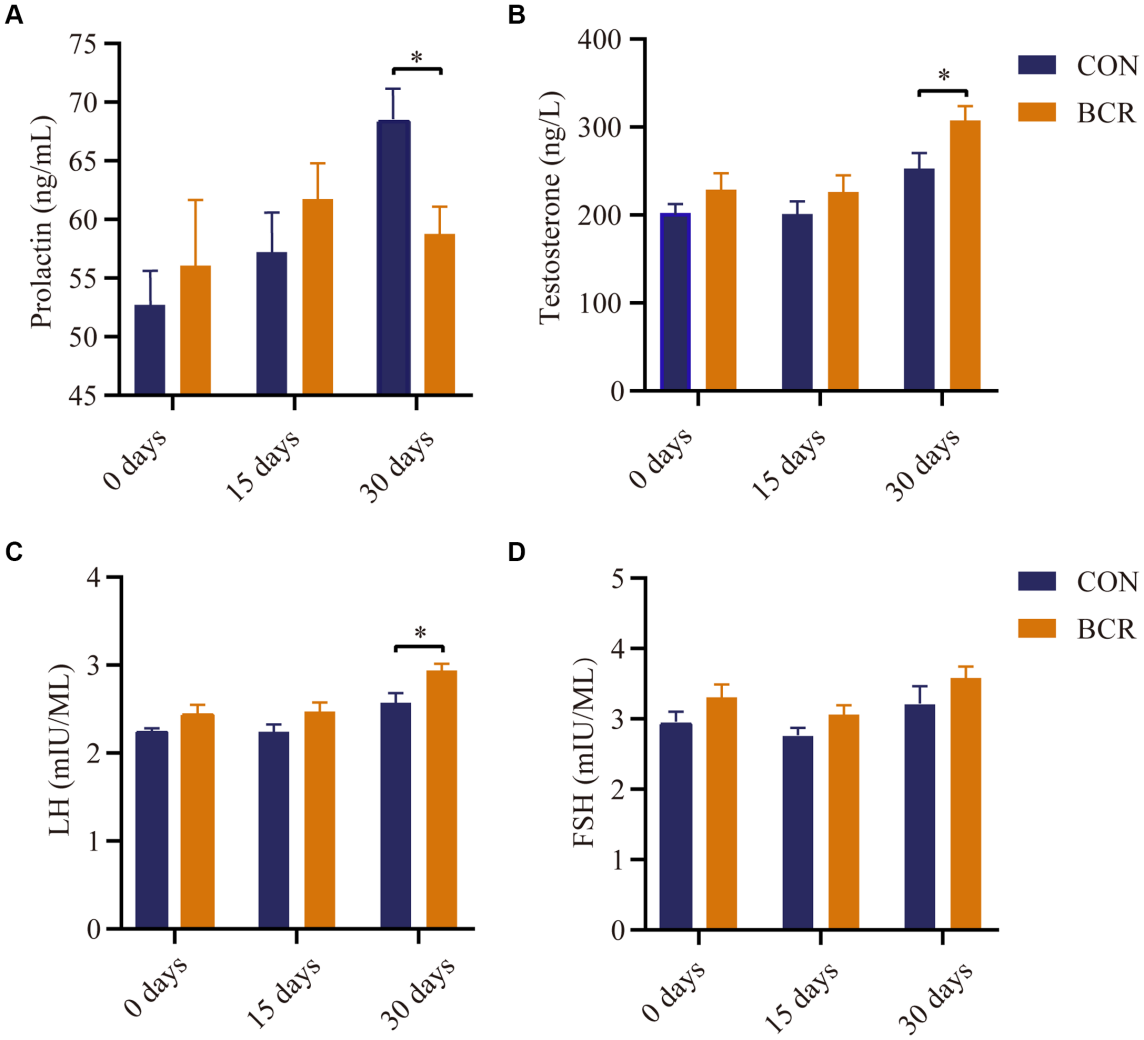
Effects of bromocriptine on serum concentration of **(A)** prolactin, **(B)** testosterone, **(C)** LH, and **(D)** FSH. Values are the mean ± standard error of the mean. FSH: follicle-stimulating hormone; LH, luteinizing hormone; CON, the control group; BCR, the bromocriptine treatment group; *p < 0.05.

There were no differences in the serum concentrations of PRL, testosterone, FSH, or LH (*p* > 0.05) on days 0 and 15 (Figure 1). Compared with the CON group, BCR significantly decreased (*p* < 0.05) the serum concentrations of PRL and significantly increased the levels of testosterone and LH (*p* < 0.05) on day 30 (Figure 1). The FSH serum level was not affected (*p* > 0.05) by treatment on day 30.

### 3.3. Testicular morphology

**Figure 2:**
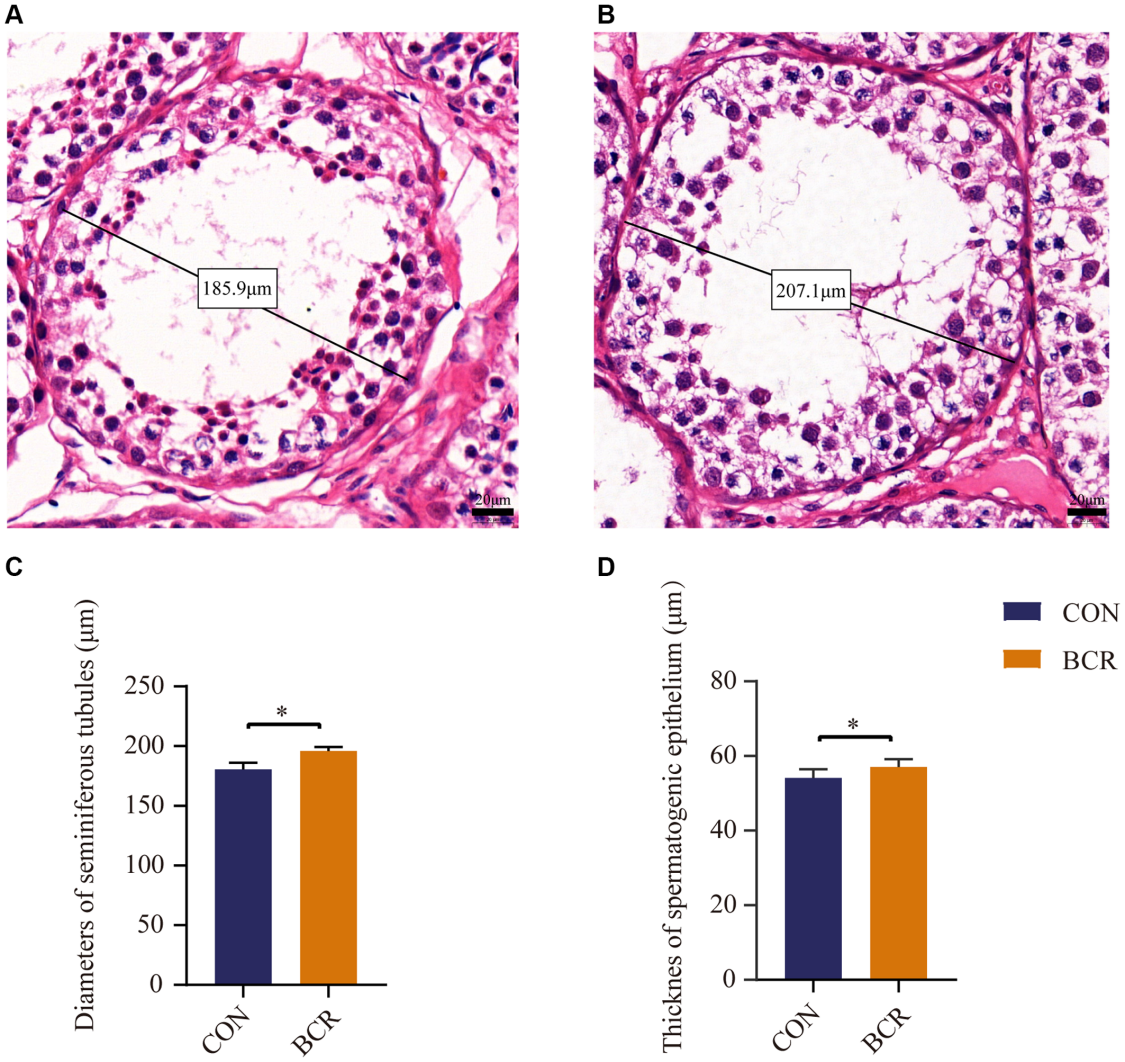
Diameter of the seminiferous tubules and the thickness of spermatogenic epithelium of goat testis from two groups with HE staining. Seminiferous tubules in the testis of **(A)** the CON group and **(B)** the BCR group depicting diameters under higher-power magnification. Scale bar = 20 μm. The diameters of the seminiferous tubules **(C)** and the thickness of spermatogenic epithelium **(D)** in goat testis. Values are the mean ± standard error of the mean. CON: the control group; BCR: the bromocriptine treatment group; *p < 0.05.

Testicular morphology of the two groups are shown in Figure 2. The mean seminiferous tubule diameter was significantly higher in the BCR group compared to that of the CON group (203.62 ± 5.55 μm vs. 180.53 ± 5.70 μm, *p* < 0.05). In addition, the thickness of the spermatogenic epithelium in the BCR group significantly greater than that of the CON group (57.06 ± 2.10 μm vs. 54.12 ± 2.30 μm, *p* < 0.05).

### 3.4. Identification of DEGs

**Figure 3:**
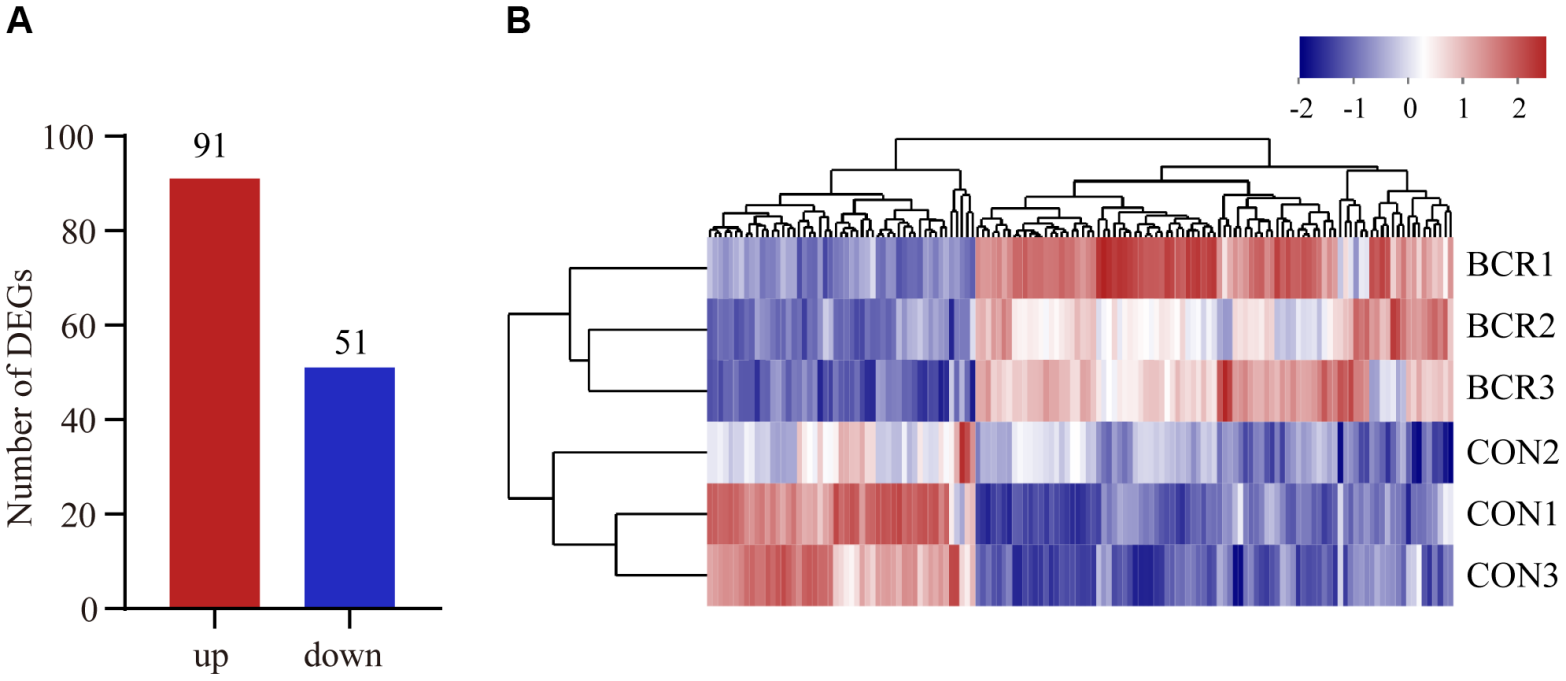
Analyzes of differentially expressed genes (DEGs) between the CON and BCR groups. **(A)** Quantity of DEGs displayed as a bar chart. **(B)** Hierarchical clustering heatmap of DEGs. Red represents upregulation and blue represents downregulation.

RNA sequencing data were analyzed for the two groups and 82,351,036–106,053,662 raw reads and 81,827,660–105,359,700 clean reads were obtained (Table 3). A total of 142 (91 upregulated, 51 downregulated) DEGs were identified in the CON vs. BCR groups (adjusted *p*-value <0.05 and |log2(fold change)| ≥ 1; Figure 3A). The details of the mRNA are presented in Supplementary Table S1. Figure 3B showed the hierarchical clustering of the differentially expressed (DE) mRNAs.

**Table 3:** Detailed information on RNA sequencing.

Sample name^a^	Raw reads	Clean reads	Raw bases (G)	Clean bases (G)	Error rate (%)	Q20 (%)	Q30 (%)	GC content (%)
CON1	87,341,224	86,768,840	13.1	13.02	0.02	98.17	94.54	49.93
CON2	83,846,588	83,289,182	12.58	12.49	0.02	98.29	94.85	49.78
CON3	84,640,348	84,063,702	12.7	12.61	0.02	98.19	94.54	48.91
BCR1	97,251,588	96,586,010	14.59	14.49	0.02	98.26	94.78	50.46
BCR2	82,351,036	81,827,660	12.35	12.27	0.02	98.23	94.68	50.04
BCR3	106,053,662	105,359,700	15.91	15.8	0.02	98.08	94.28	49.67

a CON, the control group; BCR, the bromocriptine treatment group.

### 3.5. Enrichment and functional annotation analysis of DEGs

**Figure 4:**
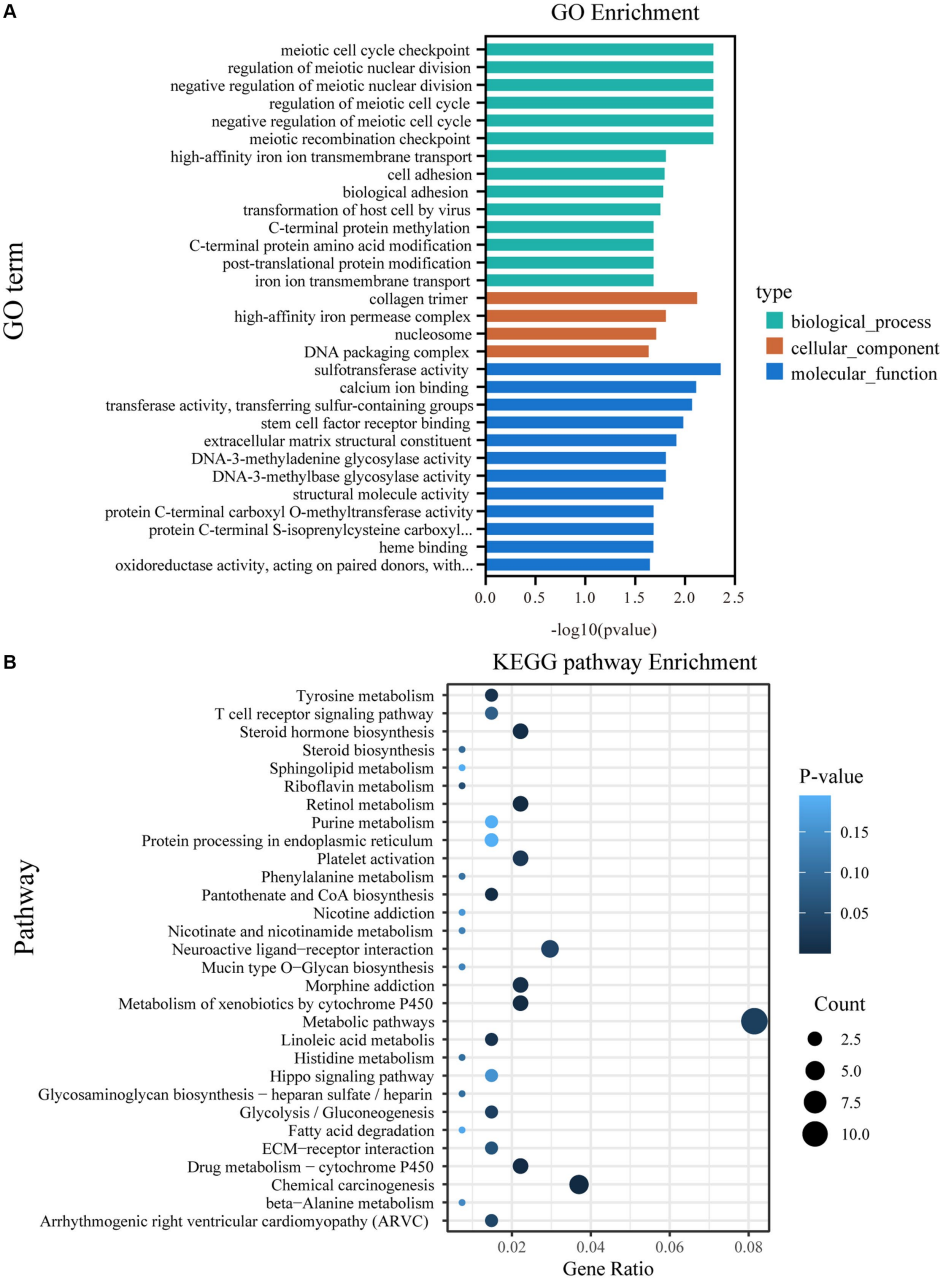
GO and KEGG pathway analyzes of DEGs. **(A)** The top 30 enriched GO terms. **(B)** The top 30 enriched KEGG pathways; GO, Gene ontology; KEGG, Kyoto Encyclopedia of Genes and Genomes; DEGs, differentially expressed genes.

According to the GO analysis, 64 GO terms were significantly enriched between the CON and BCR groups (*p* < 0.05; Supplementary Table S2). The top 30 GO terms are listed in Figure 4A. These GO terms are involved in biological processes, such as regulation of the meiotic cell cycle, transmembrane transport, cell adhesion, and post-translational protein modification. The molecular functions are associated with sulfotransferase activity, calcium ion binding, extracellular matrix (ECM) structural constituents, and structural molecule activity. KEGG analysis indicated that numerous pathways were associated with the biosynthesis of steroid hormones and spermatogenesis, including steroid hormone biosynthesis, ECM-receptor interaction, and the Hippo signaling pathway (Figure 4B and Supplementary Table S3). Notably, we found that sulfotransferase family 2 B member 1 (*SULT2B1*) and cytochrome P450 family 3 subfamily A (*CYP3A24* and *CYP3A74*) are involved in the steroid hormone biosynthesis signaling pathway.

### 3.6. Validation of DE mRNAs by quantitative real-time PCR

**Figure 5:**
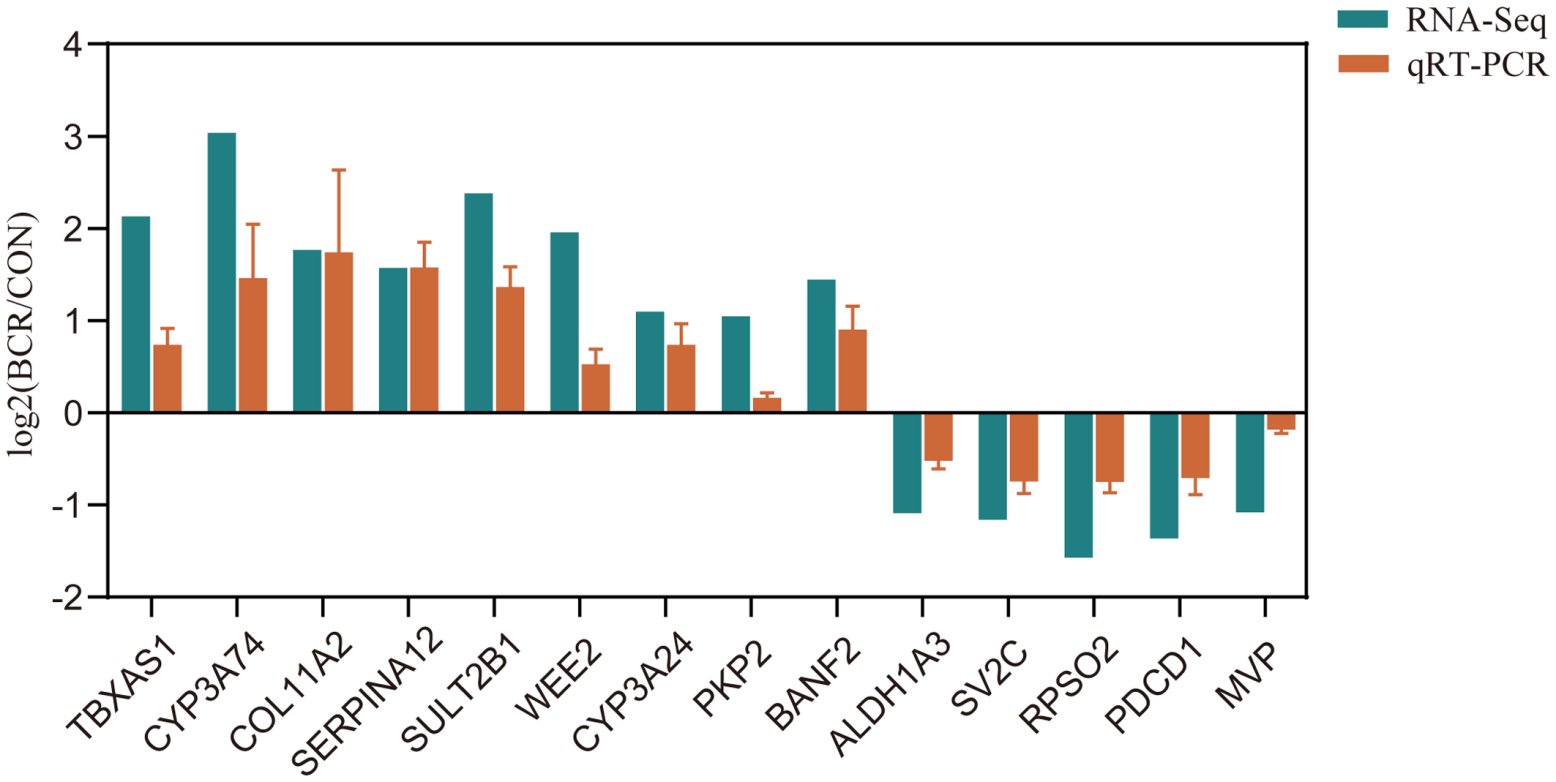
qRT-PCR validation of selected DEGs. Bar graphs present the mean ± standard error of the mean; CON, the control group; BCR, the bromocriptine treatment group; *p < 0.05.

To validate our RNA-Seq results, 14 DE mRNAs were identified: *TBXAS1*, *CYP3A24*, *CYP3A74*, *COL11A2*, *SERPINA12*, *SULT2B1*, *WEE2*, *PKP2*, *BANF2*, *ALDH1A3*, *SV2C*, *RPSO2*, *PDCD1*, and *MVP*. As depicted in Figure 5, the relative fold changes in the qRT-PCR assay were statistically significant (*p* < 0.05) and consistent with the RNA-Seq results, indicating the reliability of our RNA-Seq data.

## 4. Discussion

Testicular endocrine function is accomplished by the production of steroid hormones in Leydig cells which regulate numerous physiological processes critical for male fertility. In the present study, we investigated the changes in serum reproductive hormone levels, testicular morphology, and the transcriptome after treatment with a PRL inhibitor, and the results were consistent with our hypothesis. The serum PRL level was decreased, however, the efficacy of BCR without affecting growth. Similar to the results obtained in adult male rats (17), we found that the concentration of LH and testosterone in goats was increased, demonstrating that lower concentrations of PRL may be beneficial for testosterone production. This major relationship between PRL and LH is better described as “reciprocal,” which implies that while PRL is high and the gonadotrophins are low (22), which is consistent with our research. PRL stimulates steroidogenesis in the testes by inducing or maintaining LH receptors in Leydig cells and/or by affecting androgen biosynthesis in Leydig cells *via* steroidogenic enzymes activity (7). After BCR treatment, increased serum level of LH might improve testicular function, including testosterone production and sperm quality (23). Similar to hyperprolactinemia in humans, which can be reversed with BCR therapy, the highest secretion of PRL in rams during the summer solstice may have detrimental effects on sperm (24). Thus, regulating the serum concentration of PRL is beneficial for through increases in the concentrations of LH and testosterone.

We also observed significant changes in testicular morphology in the BCR group that may be related to the resultant increase in testosterone and/or the changes in gene expression. Maintenance of testicular function is a complex and harmonized molecular process that is regulated by numerous gene (25–27). Understanding the functional and compositional differences at the molecular level can lay the foundation for connecting these differences with testicular development and spermatogenesis in goats. In this study, we found DEGs (including *VNN2*, *HS3ST2*, *SULT4A1*, *SULT2B1*) between the treatment groups that were identified by GO functional annotation as being involved in biological processes associated with regulation of the meiotic cell cycle, transmembrane transport, cell adhesion, and post-translational protein modification. In the germ cells of the normal and hypothalamic–pituitary disconnected rams and rat testes, the expression pattern of the PRLR gene may indicate a role for PRL in the regulation of cell division, functioning as a mitotic/meiotic inducer, and/or in the cell differentiation during spermatogenesis (5, 7). Our results suggested that the DEGs (including *VNN2*, *HS3ST2*, *SULT4A1*, *SULT2B1*), which are significantly enriched in the regulation of the meiotic cell cycle may provide new insights into the mechanism of the regulation of spermatogenesis by PRL. We also saw upregulation of *SPATA25* following BCR treatment. The spermatogenesis (SPATA) family including SPATA6 (28), SPATA4 (28), SPATA17 (29), and SPATA19 (30), plays a critical role in the regulation of spermatogenesis.

In addition, the GO terms for cell adhesion, collagen trimers, and ECM structural constituents were mainly related to the ECM (31–33), including the genes *YAP1, COL11A2, EVC,* and *TM7SF2*. A KEGG analysis of DEGs also revealed the pathways of “ECM-receptor interaction” related to the ECM. As a critical portion of the wall of the seminiferous tubule, the ECM directly affects spermatogenesis and supports Sertoli and germ cells functions in the seminiferous epithelium (34), which are vital for the movement of germ cells through the blood-testis-barrier (BTB) during spermatogenesis (35). Testis is composed of the seminiferous tubules and interstitial tissues. The increased diameter of seminiferous tubule and thickness of the seminiferous epithelia may be associated with these ECM-related genes, which might be enhance the testicular function of the goats.

Interestingly, we discovered that steroid hormone biosynthesis and hippo signaling pathway were enriched and linked with testosterone secretion and spermatogenesis. As the main effector of the Hippo signaling pathway, Yes-associated protein 1 (YAP1) plays a crucial role in regulating several biological functions such as proliferation, differentiation, and cell–cell contact inhibition (36). Testosterone is a major representative androgen, which regulates the development of testes and spermatogenesis in mammals (1). Leydig cells, the primary testosterone-generating endocrine cells, are highly regulated by feedback between LH and the LH-releasing hormone (37, 38). LH binds to the luteinizing hormone receptor (LHR) on the Leydig cell membrane and activates LHR-coupled G protein, induces adenylate cyclase to increase cAMP concentration, and activates protein kinase A (PKA) and Ras in Leydig cells (39, 40). Previous studies have suggested that several genes such as steroidogenic factor 1 (*SF-1*), orphan nuclear receptor (*NUR77*), and neuropeptide Y receptor Y1 (*NPY1R*) are involved in steroidogenesis in Leydig cells (41–44). *NPY1R* belongs to the superfamily of G protein-coupled receptors and is involved in the cAMP pathway, which was downregulated by BCR treatment in the present study. Testosterone biosynthesis is mainly mediated by the steroidogenic acute regulatory protein (*StAR*), cytochrome P450 family (*CYP17A1*, *CYP11A1*), hydroxysteroid dehydrogenase (*HSD3B2*, *HSD17B3*), and LHR in Leydig cells (45). Our study revealed that the steroid hormone biosynthesis signaling pathway was enriched and *SULT2B1*, *CYP3A24* and *CYP3A74* were upregulated in the BCR group, which may explain the increased testosterone production. Previous research has also demonstrated that sulfation, desulfation, and intracellular transport of steroid hormones are the foundation for steroid hormone action (46). Cholesterol sulfate, generated by hydroxysteroid *SULT2B1*, activates the sterol regulatory element binding protein 2 (*SREBP2*), thereby promoting cholesterol biosynthesis (47). Thus, the results of RNA-Seq and qRT-PCR both indicated that genes *SULT2B1*, *CYP3A24*, and *CYP3A74*, which were differently expressed between the CON and PRL inhibitor group, might be the key factors involved in the regulation of the testis function by PRL. However, our research was primarily studied *in vivo*, the regulatory mechanisms of testosterone production need to be further explored in Leydig cells. Hence, further studies are required to delineate the function of these signature genes. Based on the results of our RNA-seq, we will investigate the roles of non-coding RNA and small RNA in PRL regulation of the testes in Leydig cells, and provide new insights into the molecular mechanisms of testicular regulation in the goat.

## 5. Conclusion

Our findings confirm that the suppression of PRL increases serum concentrations of LH and testosterone and increases the diameter of seminiferous tubules and the thickness of spermatogenic epithelium. *SULT2B1*, *CYP3A24*, *CYP3A74* genes, which are enriched in the steroid hormone biosynthesis pathway, might be essential factors involved in the PRL-mediated regulating of testis function. Overall, our study provides an understanding of the mechanisms underlying the regulation of testicular function by PRL.

## Data availability statement

The datasets presented in this study can be found in online repositories. The names of the repository/repositories and accession number(s) can be found at: https://www.ncbi.nlm.nih.gov/, PRJNA977458.

## Ethics statement

The animal study was approved by this study was conducted under the guidance of the Animal Care and Use Committee of the Hebei Agricultural University (approval number: 2023133). The study was conducted in accordance with the local legislation and institutional requirements.

## Author contributions

MC: software, formal analysis, and Writing – original draft. CD: methodology, data curation, and terms. XY: writing – review & editing. XiaoL, LZ, and SY: investigation. YZ and YL: conceptualization, resources, project administration, and funding acquisition. XianL: validation and visualization. All authors contributed to the article and approved the submitted version.
