# Supplementary material for: Prolactin inhibitor changes testosterone production, testicular morphology, and related genes expression in cashmere goats
Source: Front Vet Sci. 2023 Oct 26;10:1249189. doi: 10.3389/fvets.2023.1249189 (PMC10637432; doi:10.3389/fvets.2023.1249189)
Supplement: Supplementary file 4 [file Table_4.PDF]

Table S4

Composition and nutrient levels of TMR diet as dry matter basis

Items	Value
Ingredients (%)	
Corn straw	30
Alfalfa hay	15
Corn	37
Soybean meal	9
Wheat bran	7
$CaHPO_4$	0.2
Premix[1]	1
Sodium chloride	0.8
Nutrient levels[2]	
Metabolizable energy (MJ/kg)	9.9
DCP (%)	9.5
NDF (%)	35.33
ADF (%)	18.29
Ca (g/kg)	4.73
P (g/kg)	2.96

[1]Premix contents (per kg of diet): vitamin A, 10 456 IU; vitamin D3, 500 IU; vitamin E, 50 mg; Fe, 30 mg; Zn, 50 mg; Mn, 42 mg; Cu, 7.50 mg; Se, 0.33 mg; I , 0.70 mg; Co, 0.32 mg.

[2]Metabolizable energy and DCP were calculated values, while the others were measured values.

TMR: total mixed rations; NDF: neutral detergent fiber; ADF: acid detergent fiber; DCP: digestible crude protein
